# Molecular Pathways Underlying Projection Neuron Production and Migration during Cerebral Cortical Development

**DOI:** 10.3389/fnins.2015.00447

**Published:** 2015-12-17

**Authors:** Chiaki Ohtaka-Maruyama, Haruo Okado

**Affiliations:** ^1^Neural Network Project, Department of Brain Development and Neural Regeneration, Tokyo Metropolitan Institute of Medical ScienceTokyo, Japan; ^2^Neural Development Project, Department of Brain Development and Neural Regeneration, Tokyo Metropolitan Institute of Medical ScienceTokyo, Japan

**Keywords:** cerebral cortex, neuronal differentiation, radial migration, subplate, cortical evolution

## Abstract

Glutamatergic neurons of the mammalian cerebral cortex originate from radial glia (RG) progenitors in the ventricular zone (VZ). During corticogenesis, neuroblasts migrate toward the pial surface using two different migration modes. One is multipolar (MP) migration with random directional movement, and the other is locomotion, which is a unidirectional movement guided by the RG fiber. After reaching their final destination, the neurons finalize their migration by terminal translocation, which is followed by maturation via dendrite extension to initiate synaptogenesis and thereby complete neural circuit formation. This switching of migration modes during cortical development is unique in mammals, which suggests that the RG-guided locomotion mode may contribute to the evolution of the mammalian neocortical 6-layer structure. Many factors have been reported to be involved in the regulation of this radial neuronal migration process. In general, the radial migration can be largely divided into four steps; (1) maintenance and departure from the VZ of neural progenitor cells, (2) MP migration and transition to bipolar cells, (3) RG-guided locomotion, and (4) terminal translocation and dendrite maturation. Among these, many different gene mutations or knockdown effects have resulted in failure of the MP to bipolar transition (step 2), suggesting that it is a critical step, particularly in radial migration. Moreover, this transition occurs at the subplate layer. In this review, we summarize recent advances in our understanding of the molecular mechanisms underlying each of these steps. Finally, we discuss the evolutionary aspects of neuronal migration in corticogenesis.

## Introduction

**Figure 1:**
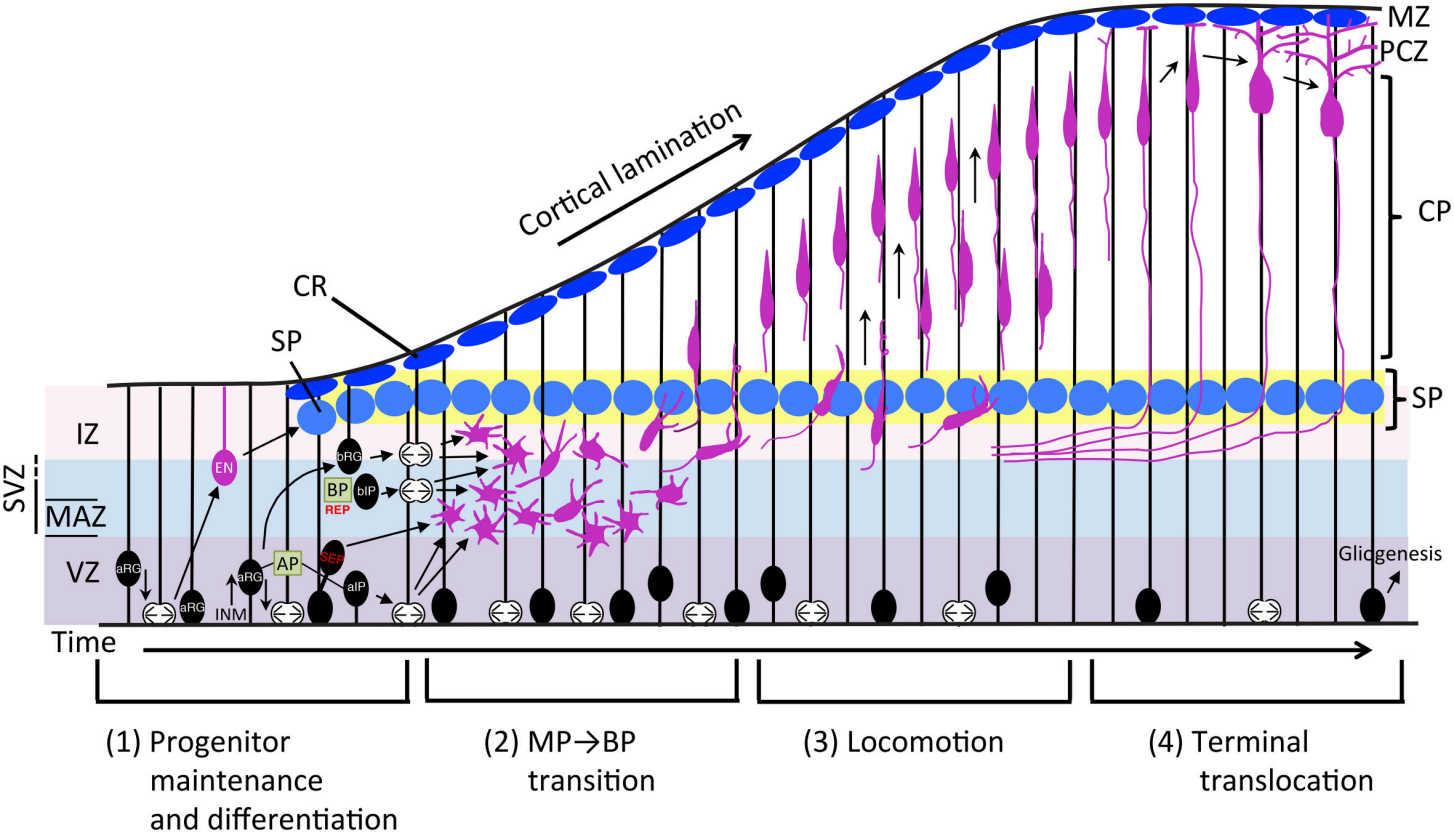
**Schematic representation of the neuronal differentiation and migration process**. The radial migration of glutamatergic neurons in the developing neocortex can be divided into four steps. (1) Neurons born from RG cells, first exhibit a MP shape and move toward the SP via MP migration where they (2) convert to BP cells. (3) After entering the CP, newborn neurons migrate toward the pial surface in locomotion mode. (4) Finally, neurons complete their radial migration by execution of terminal translocation and the initiation of maturation. aIP, apical intermediate progenitor; AP, apical progenitor; aRG, apical radial glial progenitors; bIP, basal intermediate progenitor; BP, basal progenitor; bRG, basal radial glial progenitors; CP, cortical plate; CR, Cajal-Retzius cell; EN, early born neuron; INM, interkinetic nuclear migration; IZ, intermediate zone; MAZ, multipolar cell accumulation zone; MZ, marginal zone; PCZ, primitive cortical zone; REP, rapidly exiting population; SEP, slowly exiting population; SP, subplate neuron; SVZ, subventricular zone; VZ, ventricular zone.

The mammalian neocortex is a highly organized structure underlying higher brain functions such as cognition, learning, and memory. It consists of a six-layer structure with an inside-out pattern, which is formed by radial migration of neuroblasts that continuously bypass the preceding differentiated and migrated neurons. Because neurons are born in the deeper part of the developing brain and migrate toward the pial surface, proper regulation is crucial, and impairment of this process results in various disorders such as brain malformation or psychiatric diseases. Our understanding of how this mammalian-specific complex structure is organized has advanced substantially in the last 20 years. In this review, we summarize the molecular pathways underlying how newly developed neurons travel from their birth to the terminus by dividing the process into four parts, as shown in Figure 1. Finally, we discuss evolutionary aspects of the neuronal migration mode.

## Proliferation and differentiation of neural progenitor cells

**Figure 2:**
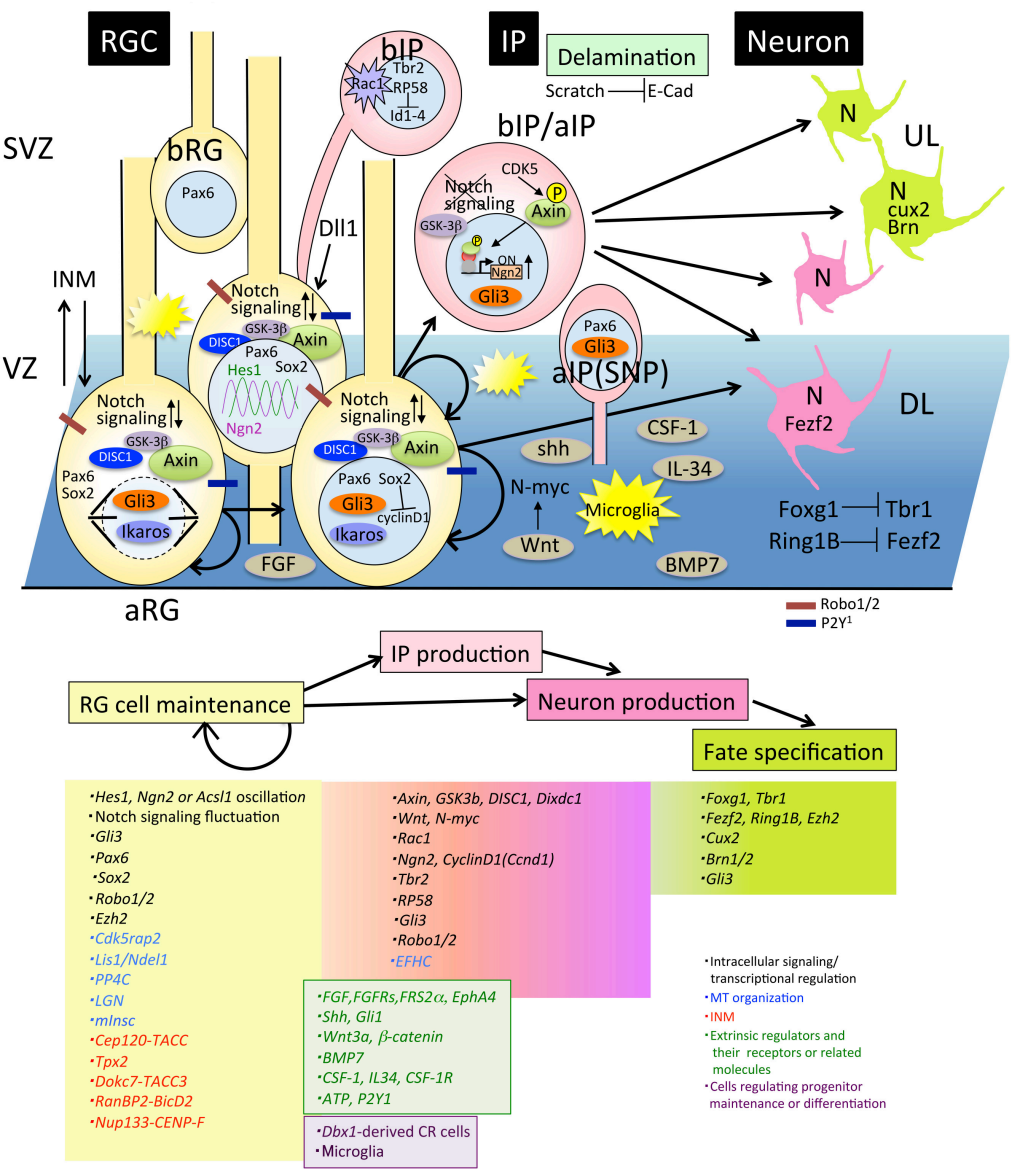
**Molecules involved in progenitor maintenance and neuronal differentiation**. Cells and molecules that regulate RG cell maintenance and IP or neuron production, fate specification, and delamination are described in Section Proliferation and Differentiation of Neural Progenitor Cells. aIP, apical intermediate progenitor; aRG, apical radial glial progenitors; bIP, basal intermediate progenitors; bRG, basal radial glial progenitors; DL, deep layer neurons; E-cad, E-cadherin; INM, interkinetic nuclear migration; IP, intermediate progenitor; RGC, radial glial cell; SNP, short neural precursor; SVZ, subventricular zone; UL, upper layer neurons.

Neural progenitor cells (NPCs) of the glutamatergic neurons of the mammalian neocortex proliferate via symmetrical division of neuroepithelial cells in the early developmental stage. During development, neuroepithelial cells become radial glia (RG) cells by expressing marker proteins that are characteristic of astrocytes, including glial fibrillary acidic protein (GFAP), astrocyte-specific glutamate transporter (GLAST), the brain lipid binding protein (BLBP), and tenascin C (TNC) around the onset of neurogenesis. RG cells have a long basal process that extends to the pial surface, and they start producing neurons by asymmetrical division while maintaining symmetrical division (Malatesta et al., 2000; Miyata et al., 2001; Noctor et al., 2001; Tamamaki et al., 2001). NPCs are classified into two subtypes based on the location of mitosis: apical progenitors (AP) and basal progenitors (BP) (Figure 1). APs are located in the ventricular zone (VZ) and include neuroepithelial cells, apical radial glia (aRG), and apical intermediate progenitors (aIPs) (Gal et al., 2006; Kawaguchi et al., 2008; Figure 2). aIPs are also called short neural precursors (SNP) which express Pax6 and divide apically (Tyler and Haydar, 2013). Basal progenitors (BPs) include basal radial glia (bRG) and basal intermediate progenitors (bIPs) which are Tbr2-positive and located mainly in the subventricular zone (SVZ). bRG are Pax6-positive RG cells preferentially located in the basal part of the SVZ called the outer SVZ (OSVZ: Smart et al., 2002) and are prominently found in gyrencephalic mammals (Fietz et al., 2010; Hansen et al., 2010; Reillo et al., 2011). Although bRG (also called oRG: outer radial glial cells or OSVZ progenitors) were also found in the developing mouse cortex as a minor population compared with those in humans and ferrets (Shitamukai et al., 2011; Wang et al., 2011b), it is thought that the diverse behaviors of bRG contribute to variations in the cortical structure between mammalian species (Gertz et al., 2014). Compared with neuroepithelial cells that divide only symmetrically in the proliferative state, aRG produce two progenitors by symmetrical division, or one progenitor and one neuron or intermediate progenitor (IP) by asymmetrical division in the neurogenic stage. bIPs are Tbr2-positive and neuron-committed progenitors that divide in the majority of cases once, or in a minority of cells twice, to produce neurons suggesting its role in the amplification of the progenitor pool (Haubensak et al., 2004; Noctor et al., 2004; Kowalczyk et al., 2009). bRG, another population of transient-amplifying cells, are more prominent in the gyrencephalic cortex compared with that in rodents, suggesting that IPs and bRG may contribute to amplifying neuron numbers, the expansion of cortical area, and gyrification during evolution (Lui et al., 2011). In addition, novel progenitor cells, called subapical progenitors (SAP) were described recently (Pilz et al., 2013).

Thus, a variety of progenitors proliferate and differentiate into neurons during the cortical developmental stage, suggesting that an accurate balance between the proliferation state and differentiation into neurons is critical for determination of the final number of cortical neurons (Caviness et al., 1995). In this regard, experiments showed that a disturbance of the balance between self-renewal and differentiation of mouse NPCs promotes cortical expansion (Chenn and Walsh, 2002). In this study, transgenic mice expressing a stabilized β-catenin in NPCs develop enlarged brains with increased cerebral cortical surface area and folds resembling sulci and gyri of higher mammals (Chenn and Walsh, 2002), suggesting that the precise regulation of the proliferative state of either NPC maintenance or NPC differentiation maybe a critical factor for regulating cerebral cortical size during evolution. How this regulation is orchestrated has been a topic of interest, and many researchers have been investigating it from various standpoints. We will summarize the mechanisms of maintenance and differentiation of the NPCs from the intracellular and extracellular standpoints (Figure 2).

### Regulation of intracellular signaling of neural progenitor cells

Regarding intracellular signaling of NPCs (Figure 2), studies have revealed that Notch signaling fluctuation plays a critical role in the maintenance of the progenitor state (Kageyama et al., 2008; Shimojo et al., 2008). Their findings originated from the discovery that the expression of the bHLH factor *Hes1* oscillates with a duration of about 2-3 h in many cell types (Hirata et al., 2002). In NPCs, *Hes1* oscillation drives the oscillatory expression of *Neurogenin 2 (Ngn2), Ascl1*, and *Dll1*, a key ligand for activating Notch signaling (Shimojo et al., 2008; Imayoshi et al., 2013). The oscillatory expression between *Ngn2* or *Ascl1* and *Dll1* in the complementary phase leads to mutual activation of Notch signaling within neighboring progenitors, and enables the progenitors to maintain the proliferative state of NPCs. Once this oscillation is diminished by sustained expression of proneural factors, progenitor cells differentiate into different neuronal and glial subtypes based on proneural factor that shows sustained expression levels (Imayoshi et al., 2013). How oscillatory expression levels of *Hes1, Ascl1, Ngn2*, and *Dll1* influence the fate of progenitor cells in relation to developmental time is still unknown. Although epigenetic modification of the NPC genome could be considered as a candidate, identification of the downstream genes of these oscillation will reveal the molecular mechanisms of cell fate specification in future studies.

Notch signaling fluctuation in NPCs is also supported by the gene expression profiles of a large number of single progenitor cells at the mid-embryonic stage (Kawaguchi et al., 2008). They classified progenitors in three subclasses according to their gene expression profiles and found that APs exhibit highly variable expression patterns of Notch signaling related genes. Attenuation of Notch signaling in APs immediately led to differentiation of APs into nascent IPs. Interestingly, a recent report revealed that IPs provide feedback to the RG progenitors by serving as a source of Dll1 via dynamic and transient processes that directly interact with RG (Nelson et al., 2013). This feedback regulation may be involved in maintaining the RG progenitor pool by activating Notch signaling in RG cells (Figure 2). As for the regulators for Notch signaling, Robo1, and Robo2 receptors that are known axon guidance regulators, have been reported to modulate the transition between RG cells and IPs through activation of the Notch effector *Hes1* (Borrell et al., 2012). This study suggested that some regulators have multiple functions in cortical development including neurogenesis and neural circuit formation.

Sox2, a member of the high-mobility group box transcription factors, is highly expressed in RG cells and is essential for maintaining their self-renewal state (Hutton and Pevny, 2011). Recently, the molecular mechanism has been revealed by which Sox2 negatively regulates genes promoting NPC proliferation including *Cyclin D1* using single cell RNA-seq experiment of *in utero* electroporation (IUE) cortex. Upon differentiation to IP cells, upregulated Ngn2 repressed Sox2 followed by cyclin D1 de-repression, and promote IP proliferation (Hagey and Muhr, 2014).

Other factors that are involved in progenitor pool maintenance include Axin (Fang et al., 2013), Disrupted in Schizophrenia 1(DISC1) (Mao et al., 2009), and Rac1 (Leone et al., 2010). Axin is a scaffold protein for many signaling proteins, including GSK-3. An increase in the expression level of Axin in progenitor cells leads to the transient amplification of IPs without affecting the RG pool. In this state, Axin localized in the cytoplasm with GSK-3 as a binding partner contributes to the self-renewal and IP amplification of aRG. As the neurogenic stage proceeds, Axin is phosphorylated by cyclin dependent kinase 5 (CDK5) and translocated into the nucleus with β-catenin as a binding partner, which is followed by a shift to neuronal differentiation. This function of Axin is independent of the canonical Wnt signaling pathway. These results suggested a novel role of Axin in IP expansion during evolution (Fang et al., 2013). Regarding GSK-3, it has been reported that the deletion of GSK-3 signaling by genetic elimination of all isoforms resulted in massive hyperproliferation of neural progenitors and markedly suppressed generation of both IPs and postmitotic neurons (Kim et al., 2009). DISC1, originally identified as a Schizophrenia- related gene (Blackwood et al., 2001) plays an important role in many aspects of neural development. For progenitor maintenance, DISC1 regulates RG cell proliferation via inhibition of GSK-3 by directly binding and modulating the canonical Wnt pathway together with its binding protein Dixdc1 (Mao et al., 2009; Singh et al., 2010). Taken together, this evidence indicates that GSK-3 signaling is essential for maintenance of the neural progenitor pool during cortical development. Meanwhile, as for Wnt-signaling, it has also been reported that N-myc is a downstream target that promotes IP production (Kuwahara et al., 2010).

Rac1, a small G-protein that is a member of the Rho-GTPase family, has been implicated in regulating the proliferation and differentiation of stem cells of various tissues (Benitah et al., 2005; Chrostek et al., 2006). A forebrain-specific loss of Rac1 leads to reduction in proliferation in a SVZ-progenitor (bIP)-specific fashion, a concomitant increase in cell cycle exit and premature differentiation (Leone et al., 2010), which suggests that Rac1 activity is crucial for maintenance of the progenitor state of bIPs.

### Fate specification of neural progenitor cells

The neuron production stage includes another important step of neuronal subtype specification for which many transcription factors are involved. FoxG1 is a transcriptional repressor that is strongly expressed in progenitors and functions as a repressor of Cajal Retzius (CR) cell competency (Hanashima et al., 2004) as well as a regulator of NPC self-renewal, IP expansion, and the timing of neurogenesis (Shen et al., 2006; Siegenthaler et al., 2008; Fasano et al., 2009). It has been reported that *Foxg1* is necessary and sufficient for inducing deep-layer neurogenesis and that it switches the transcriptional program to acquire upper layer neuron identity through direct repression of transcription expression of T-box brain 1 (Tbr1) transcription factor, an early born postmitotic neuronal marker (Kumamoto et al., 2013; Toma et al., 2014). This suggests that expression of a single transcription factor may enable neural progenitors to alter their intrinsic character.

The transcription factors Fezf2 and Cux2 are neuronal subtype markers expressed in deep and superficial layer neurons, respectively, and they are critical for fate specification (Nieto et al., 2004; Chen et al., 2005). They are also expressed in NPCs in VZ, which has led to a debate about the existence of fate-restricted progenitors in Cux2- or Fezf2-Cre driver mouse lines (Franco et al., 2012; Guo et al., 2013). Although a recent report using the mosaic analysis with double marker (MADM) system (Zong et al., 2005; Hippenmeyer et al., 2010) demonstrated unitary production of deep and superficial layer neurons by individual NPCs (Gao et al., 2014), this dispute remains unresolved.

Other transcription factors involved in neurogenesis regulation include Pou3fs (Brn1, Brn2) and Gli3. Brn1/2 is a crucial regulator of the production of upper-layer neurons, and its expression in VZ progenitors is essential for the transition from early to mid-neurogenesis (Sugitani et al., 2002; Dominguez et al., 2013). Gli3 is a transcription factor in the Hedgehog (Hh) pathway, the loss of which in RG cells results in decreased production of IPs and prolongs the production of deeper cortical neurons, suggesting that Gli3 is required for both the generation and maintenance of IPs and fate specification of IP-originating superficial neurons (Wang et al., 2011a).

Besides transcription factors, it has been shown that chromatin regulators are also critical for the fate specification of NPCs (a review, see Tyssowski et al., 2014). Ring1B is a component of the polycomb group (PcG) complex 1(PCR1) proteins and functions as a repressor of transcription via trimethylation of residue Lys27 of histone H3 (H3K27me3). Reports from the Gotoh research group have shown that Ring1B is essential not only for shifting the neurogenic state to an astrogenic fate (Hirabayashi et al., 2009) but also for terminating the production of deep-layer neurons through direct repression of *Fezf2* promoter activity (Morimoto-Suzki et al., 2014). They have also shown that depletion of *Ezh2*, which is a component of the polycomb group (PcG) complex 2(PCR2), exhibited the same phenotype of prolonged neurogenic phase of NPCs and delayed onset of the astrogenic phase, as depletion of Ring1B at the same stage (E12.5) (Hirabayashi et al., 2009). However, depletion of *Ezh2* before the onset of neurogenesis results in the opposite effects, that is, accelerate differentiation and early onset of astrocyte production (Pereira et al., 2010). These results suggest that Ezh2 may independently regulate the major developmental transitions in cortical progenitor cells: expanding neuroepithelial cell by self-renewing, producing neurons of different laminar fates, and switching from neurogenesis to gliogenesis. Ikaros, another modulator of the chromatin-remodeling complex, is expressed in NPCs at highest levels during the early stage of neurogenesis, and its expression decreases as development proceeds. Sustained expression of Ikaros results in prolonged production of deep-layer neurons, supporting its role in fate determination of deep-layer neurons via chromatin regulation (Alsiö et al., 2013).

### Progenitor maintenance and microtubule (MT) organization

Microtubules (MTs) are an important component of cytoskeletons and are vital for the organization of the centrosome and mitotic spindle, which are also crucial for the maintenance of NPCs. EFHC1 is a protein containing a single EF-hand motif, a Ca^2+^ binding domain, which directly interacts with α-tubulin. Mutation in the gene encoding this protein causes juvenile myoclonic epilepsy (Suzuki et al., 2004). Functional analysis of EFHC1 using rat developing neocortex revealed that it is essential for cell cycle exit of NPCs via the assembly and function of mitotic spindle. Impairment of this gene affects mitotic spindle formation and M-phase progression by microtubule bundling defects and increased apoptosis (de Nijs et al., 2009).

Cdk5rap2 is localized at the centrosome of neural progenitors, and loss of this protein causes a failure in the maintenance of the neural progenitor pool by increased cell cycle exit followed by premature neuronal differentiation (Buchman et al., 2010). The microtubule-binding protein Hook3 is recruited to pericentriolar satellites through an interaction with Pericentriolar Material 1 (PCM1). Disruption of the Hook3-PCM1 interaction impairs maintenance of the neural progenitor pool (Ge et al., 2010). This suggests that regulators of centrosome dynamics are also important for progenitor maintenance. In addition, the regulation of mitotic spindle orientation is also important for symmetric division and thereby, progenitor maintenance (Yingling et al., 2008). It has been reported that the mitotic spindle of RG cells orients almost parallel to the ventricular surface in both proliferative and neurogenic stages. Only a fraction of RG cells that adopt divisions with oblique and vertical spindle orientations preferentially generate bIPs and bRG during the neurogenic stage (Konno et al., 2008; Shitamukai et al., 2011). A disturbance of mitotic spindle orientation by knocking out *LGN* (G protein regulator) gene or manipulation of the mouse *Inscuteable*(*mInsc*) gene expression level leads to a disruption of the balance between proliferation and differentiation of NPCs (Konno et al., 2008; Postiglione et al., 2011). Lis1, its binding partner Ndel1, and dynein form a complex that is also required for maintaining spindle orientation perpendicular to the ventricular surface and NPC proliferation (Yingling et al., 2008). Recently, it has been reported that the protein phosphatase PP4c regulates spindle orientation in early cortical progenitor cells by dephosphorylating Ndel1, thereby enabling complex formation with Lis1 to form a functional spindle orientation complex (Xie et al., 2013). These lines of evidence demonstrate that regulation of mitotic spindle orientation is one of the key molecular mechanisms for progenitor maintenance and the transition between symmetric and asymmetric cell division.

### The behavior of neural progenitor cells in the ventricular zone

Now we turn our attention to the motion of NPCs. It has been long known that nuclei of progenitor cells exhibit cell-cycle dependent oscillatory movement known as interkinetic nuclear migration (INM), also called elevator movement (Sauer and Walker, 1959; Fujita, 1962, 1963). Although the molecular mechanisms and the biological meaning of this movement are not well understood, recent studies provide insights into the molecular mechanisms of INM. The functional roles of both microtubule and actomyosin motor proteins in INM were identified first (Tsai et al., 2005, 2010; Norden et al., 2009; Schenk et al., 2009). Next, the involvement of other proteins in INM regulation was reported. Centrosomal protein of 120 kD (Cep120) is a centrosomal protein expressed in NPCs and knockdown of Cep120 results in impairment of INM through interactions with transforming acidic coiled-coil proteins (TACCs) (Xie et al., 2007). Hook3, mentioned above, is involved in regulation of INM, suggesting that INM is an important behavior of NPCs for proper neurogenesis of the mammalian neocortex, and it has been reported in other neurogenic systems (Murciano et al., 2002; Del Bene et al., 2008). Tpx2, a microtubule-associated protein, has been identified as an essential protein for apical nuclear migration during G2 phase (Kosodo et al., 2011). Dock7, a member of the DOCK180 superfamily of a distinct class of Rac/Cdc42 GTPase guanine nucleotide exchange factors (GEFs), regulates INM by interacting with TACC3 (Yang et al., 2012b). Meanwhile, it has been shown that dynein recruitment to the nuclear pore is required for apical nuclear migration through “RanBP2-BicD2” and “Nup133-CENP-F” pathways (Hu et al., 2013).

Moreover, a recent study revealed the biological role of INM. Using TAG-1 knockdown, which leads to loss of the basal processes of RG cells and *in toto* imaging, it was shown that proper INM is critical for preventing overcrowding of progenitor cells and for facilitating the smooth departure of the differentiated cells from the VZ (Okamoto et al., 2013).

### Extrinsic regulators of neurogenesis

Besides the cell-autonomous regulatory mechanisms described above, extrinsic factors are also involved in the regulation of NPC maintenance. Several growth factor signaling pathways including for fibroblast growth factor (FGF), sonic hedgehog (shh), Wnt, bone morphogenetic proteins (BMPs), colony stimulating factor-1 (CSF-1), and interleukin (IL) 34 are involved in the regulation of progenitor self-renewal and differentiation. Targeted disruption of the docking protein FRS2α, a major mediator of FGF signaling, leads to severe impairment of cerebral cortical development with thinner cerebral cortices than wild-type (WT) cells, reduced proliferation and differentiation of Tbr2- positive bIPs (Yamamoto et al., 2005). Genetic disruption of all three FGF receptors (FgfRs) leads to attenuation of Notch signaling and precocious production of bIPs followed by premature termination of neurogenesis (Rash et al., 2011). Recently, it has been reported that this function in progenitor maintenance of FGF occurs in cooperation with EphA4, a member of the receptor tyrosine kinase superfamily (Chen et al., 2015). Shh, known as a regulator of early central nervous system (CNS) development, also regulates progenitor proliferation through upregulation of Gli1, which is a zinc finger transcriptional factor and a mediator of Shh signaling (Dahmane et al., 2001). Targeted disruption of *shh* in the mouse dorsal pallium leads to small cerebral cortices at embryonic day (E)18.5, which was caused by impairment of cell cycle exit and reduced proliferation of NPCs (Komada et al., 2008). This regulatory function of shh in neurogenesis is cooperative with Notch signaling (Dave et al., 2011). As mentioned in Section Regulation of Intracellular Signaling of NPCs, Wnt also regulates IP production. Ectopic Wnt3a expression in the developing cortex causes cortical dysplasia and neuronal heterotopias (Munji et al., 2011). The authors found that Wnt3a promotes expansion of RG and differentiation of IPs. These results suggested that the Wnt-β–catenin pathway regulates both RG self-renewal and IP differentiation (Munji et al., 2011). Bmp7 null embryos exhibited microcephaly by reduced cortical plate thickness. It has been revealed that Bmp7 is required for the proliferation potential of NPCs (Segklia et al., 2012). The CSF-1 receptor (CSF-1R), known in CNS microglial development, has been revealed as another regulator in progenitor maintenance. *Csfr-/-* mice displayed increased proliferation, apoptosis of NPCs, and reduced differentiation of specific excitatory neuronal subtypes (Nandi et al., 2012). This result suggested that CSF-1 and IL-34, ligands of CSF-1R, suppress self-renewal potential of RG cells and production of IPs to maintain the balance between proliferation and differentiation.

Cells and meninges also function as signaling centers regulating NPC proliferation. The distribution of a subtype of CR cells (*Dbx1*-derived CR cells) influences the proliferation and differentiation of progenitor cells in the VZ (Griveau et al., 2010). Thus, CR cells provide certain information to NPCs for their proliferative state via secretion of signaling molecules. Loss of meninges in the forebrain by *Foxc1* mutation results in the reduction of retinoic acid (RA) secretion and impairment of switching from symmetric to asymmetric division, thus leading to a decrease in neuron and IP production (Siegenthaler et al., 2009). Additionally, tangentially migrating transient glutamatergic neurons that are generated by *Dbx1* positive progenitors at the pallial/subpallial boundary (PSB) at E12.5 contribute to maintain the neocortical progenitor pool (Teissier et al., 2010, 2012). These studies highlight the major involvement of such extrinsic regulators in NPC maintenance and differentiation.

ATP signaling and calcium waves are also involved in the regulation of NPCs. Spontaneous calcium waves that are dependent on connexin hemichannels and P2Y_1_ ATP receptors propagate through RG cells in the VZ and regulate neuron production (Weissman et al., 2004) and are essential for migration of IPs to the SVZ (Liu et al., 2008). Another class of novel participants among the regulators of progenitor cell maintenance is microglia, resident macrophages in the brain (Cunningham et al., 2013). Activated microglia colonize the proliferative zones of the developing rat and primate forebrains, and the manipulation of microglia cell numbers significantly affects the number of NPCs. Microglial surveying and its crucial role in eliminating injured neurons in adult brains has been known; excessive microglial activation was observed in autism spectrum disorder (ASD) (Nimmerjahn et al., 2005; Tetreault et al., 2012; Suzuki et al., 2013). However, Cunningham et al. showed that microglia also play an important role in normal cortical development during embryogenesis by eliminating NPCs at the end of cortical neurogenesis and, therefore, they may contribute to terminate neurogenesis (Cunningham et al., 2013).

## Multipolar cell to bipolar cell transition

**Figure 3:**
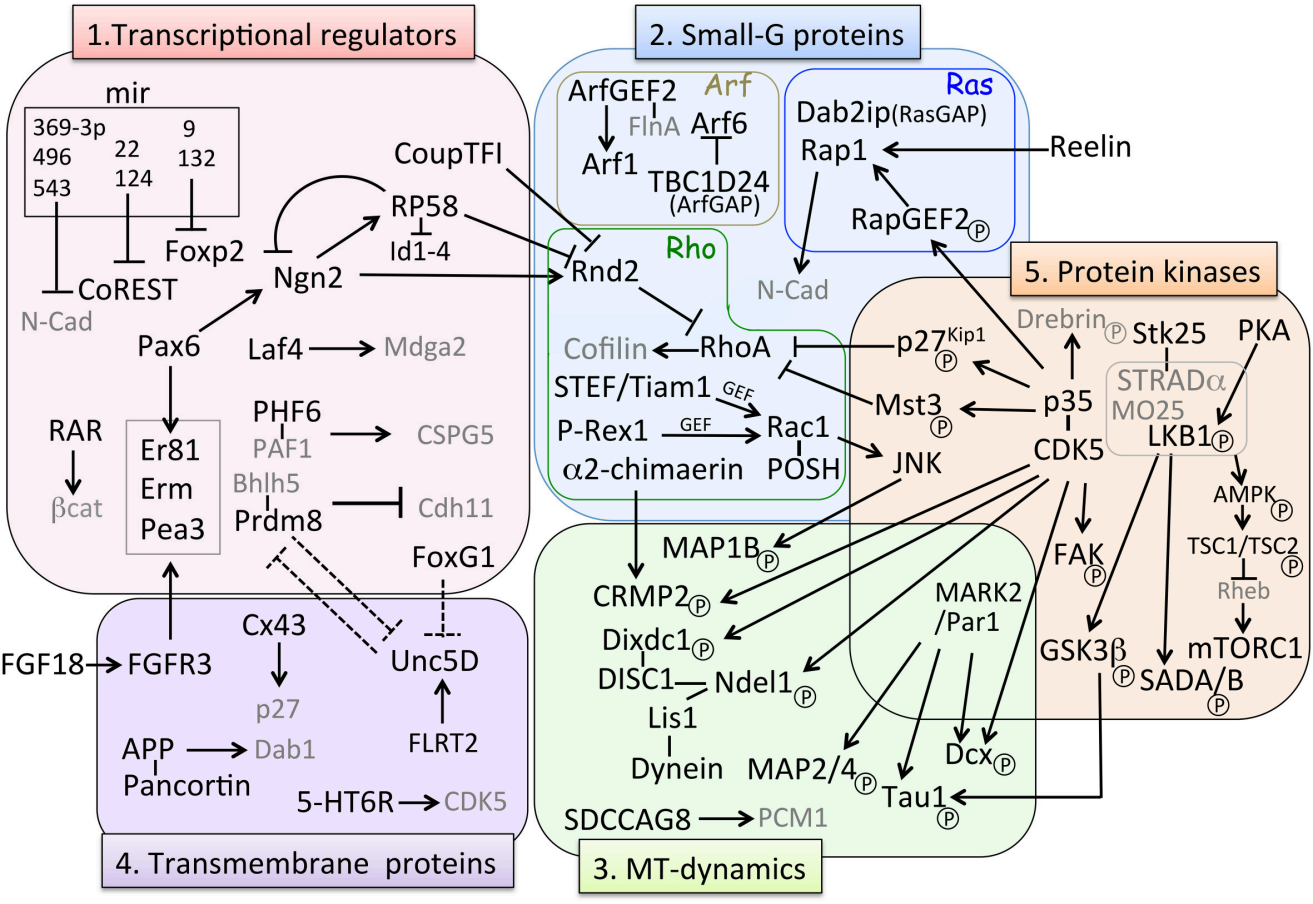
**Molecular pathways involved in MP-BP transition**. Factors involved in the MP-BP transition can be categorized in the following five groups based on their molecular function: (1) transcriptional regulators, (2) small-G proteins, (3) microtubule (MT)-dynamics, (4) transmembrane proteins, and (5) protein kinases. Black arrows (**→**) represents positive regulation of either “activated,” “stabilized,” or “phosphorylated” target factors; ⊣ indicates either “repression of transcription” or “inhibition of activity” of target factors. Dashed lines indicate putative relationships inferred from the experimental data. Factors in gray characters are effectors of their partner molecules but they do not belong to each category. BP, bipolar; MP, multipolar; MT, microtubule.

Newly born late-born neurons finally depart the VZ and start the journey to their final destinations. During radial migration, cell shape and migration mode change markedly. After differentiation, newborn neurons exhibit a multipolar (MP) shape with multiple neurites and migrate in a MP migration mode in random directions (Tabata and Nakajima, 2003). For MP cell migration, it has been reported that two distinct populations in terms of their migrating behaviors exist (Tabata et al., 2009). One is the slowly exiting population (SEP), in which postmitotic MP migrating cells stay in the lower part of the SVZ called the MP cell accumulation zone (MAZ). The other is the “rapidly exiting population (REP),” which migrate rapidly into the SVZ/intermediate zone (IZ) and undergoes further cell division, then converts to MP cells. REP includes bIPs, Olig2-positive glial progenitors, and probably bRG. Whereas, the SEP stays in the MAZ but enters the cortical plate (CP) faster than the REP and contributes to the production of superficial neurons as well as the REP (Tabata et al., 2009; Figure 1). This study indicated that the migration behavior of the direct progeny of asymmetric division and IPs are different although both of them exhibit MP shapes. For delamination of differentiated cells from the apical surface of the VZ, Scratch 1, and 2, members of the snail super-family of transcription factors, are involved in this regulation through the suppression of E-cadherin (Itoh et al., 2013). After delamination, MP cells convert to bipolar (BP) cells for locomotion. Neuronal polarization of newborn neurons (neuroblasts) *in vivo* occurs during this step, starting with extension of a thin axon, and one selected neurite became a thick leading process. Time-lapse imaging of cultured slices of electroporated brains showed that axon extension occurs prior to the formation of the leading processes for majority of MP cells (Hatanaka and Yamauchi, 2013). Additionally, this axon specification is dependent on TAG-1-mediated contact between immature neurites and axons of early born neurons (Namba et al., 2014). Neuronal polarization in dissociated primary cultured neurons is defined as axon specification from multiple neuritis. In contrast, leading process formation to convert a BP cell is a critical polarization step *in vivo* (Takano et al., 2015). Many gene knockouts or knockdowns of cortical development exhibit phenotypes of either delay or failure of the MP-BP transition, suggesting that this regulatory mechanism is crucial for the radial migration process. Many genes have been reported to be involved in this regulation. These can be classified roughly into five categories (Figure 3): (1) transcriptional regulators, (2) small GTP-binding proteins, (3) proteins related to MT dynamics, (4) receptors and other membrane proteins, and (5) kinases. Nevertheless, the downstream effectors are mostly involved in cytoskeletal regulation. We will summarize recent findings for each category (Figure 3).

### Transcriptional regulators involved in the multipolar-bipolar transition

Neurogenin 2 (Ngn2), a proneural transcription factor responsible for glutamatergic neuronal differentiation from NPCs, has been found to also play an important role in the MP-BP transition via direct transcriptional activation of the small GTP-binding protein Rnd2, an atypical Rho-GTPase protein that inhibits RhoA activity and regulates actin cytoskeleton (Heng et al., 2008). Transcriptional repressor RP58 (also known as zfp238, znf238, and zbtb18) is not only another downstream target gene of Ngn2 (Xiang et al., 2012) but also an upstream regulator repressing Ngn2 transcription by negative feedback regulation (Ohtaka-Maruyama et al., 2013). Meanwhile, Rnd2 transcription is directly repressed by RP58 (Heng et al., 2015) and CoupTF-I, a nuclear orphan receptor (Alfano et al., 2011), suggesting that the expression level of Rnd2 is critical for the MP-BP transition (Table 1, Figure 3). We have been studying the functional roles of RP58 and have found that this transcriptional repressor plays critical roles in neuronal migration as well as neuronal differentiation (Okado et al., 2009; Ohtaka-Maruyama et al., 2012, 2013). We summarize the transcriptional regulators involved in the MP-BP transition in Table 1.

**Table 1:** **A summary of transcriptional regulators involved in the MP-BP transition**.

**Transcriptional regulator**	**Gene manipulation method**	**Examined developmental stage**	**Migration defect**	**Related gene, protein**	**References**
*Neurogenin2*	*Ngn2(–/–)* (LOF)	E14.5→4 DIV	Impairment of CP entering	RhoA	Hand et al., 2005
(*Ngn2*)	*Ngn2^*Flox*∕*Flox*^*+ Cre plasmid (LOF) *Ex vivo* electroporation Slice culture			*Rnd2*	Heng et al., 2008
*RP58*	*RP58^(−∕−)^*(LOF) *RP58^*Flox*∕*Flox*^* + Cre plasmid(LOF) IUE	E14.5→E17.5, E19.5	Impairment of CP entering Accumulation of MP cells	*Ngn2* *Rnd2*	Ohtaka-Maruyama et al., 2013 Heng et al., 2015
*Coup-TFI*	*Coup-TFI(–/–)* (LOF)	E14.5→4DIV, E18.5, P8	Impairment of CP entering	*Rnd2*	Alfano et al., 2011
	*Coup-TFI^*Flox*∕*Flox*^* + Cre plasmid (LOF)		Abnormal MP cell morphology		
	IUE, *Ex vivo* electroporation Slice culture		Defective axonal elongation of CPNs		
*FoxG1*	*CAG-FoxG1-IRES-EGFP(GOF)*	E13.5→E16.5, E19.5	Delay of the radial migration (GOF) Altering the lamina fate (GOF)	Unc5D	Miyoshi and Fishell, 2012
	*Ngn2 CreER, FoxG1-C:Flpe/-, R26R-CAG-FRTstop-EGFP reporter + Tmx (LOF)*	Tmx E11.5→E14.5 Tmx E13.5→E16.5 Tmx E15.5→E18.5	Impairment of CP entering (LOF)		
	*NeuroD1-mCherry IRES CreER* (Late LOF) IUE	E12.5 Tmx→E16.5 →E19.5	Late LOF does not affect postmigratory populations		
*Laf4/Aff3*	sh-RNA (LOF)	E14.5→6 DIV	Impairment of CP entering	*Mdga2*	Moore et al., 2014
	*Laf4-HA-IRES-GFP* (GOF)				
	IUE, Slice culture				
*PHF6*	sh-RNA (LOF)	E14.5→E17.5, 19.5, P6	Delay of the radial migration	PAF1	Zhang et al., 2013a
	IUE		Accumulation of MP cells	NGC/CSPG5	
			White matter heterotopia		
*Prdm8*	*CAG-Prdm8* plasmid (GOF)	E12.5→P5	Impairment of CP entering (GOF)	Unc5D	Inoue et al., 2014
	sh-RNA (LOF)	E14.5→E17, E17.5	Accumulation of MP cells (GOF)		
	IUE		Premature BP transition (LOF)		
			Altering the laminar fate (LOF)		
*Erm, Er81* (Pea3-Ets family)	DN-plasmid (LOF) IUE	E13.5→E16.5	Impairment of CP entering	FGF18, FFGFR	Hasegawa et al., 2004
miR-9, 132 (Target: FoxP2)	*FoxP2-3*′UTR	E13.5→E18.5, P15	Delay of the radial migration	*FoxP2*	Clovis et al., 2012
	*FoxP2*- Δ -3′UTR (GOF)		White matter heterotopia		
	FoxP2-3′UTR-MT1+2+3 (GOF)				
	IUE				
miR-379-410 cluster	NeuroD1-miRNA plasmid (LOF)	E13.5→E17.5	Impairment of CP entering (LOF)	*N-cadherin*	Rago et al., 2014
(Target: N-cadherin)	NeuroD1-anti-miR LNAs plasmid (GOF) IUE		Enhancement of the radial migration (GOF)		
miR-22,124 (Target: CoREST)	*Dicer* ^*Flox*∕*Flox*^ + *NeuroD:Cre-GFP* plasmid	E14.5→3DIV, E17.5 →E18.5, P2	Delay of the radial migration (GOF)	CoREST Dcx	Volvert et al., 2014
	miR expression plasmid (LOF) antagomiR (GOF) IUE, Slice culture	E14.5→E16.5→1DIV →time-lapse	Accumulation of MP cells (GOF)		
Retinoic acid receptor (RAR)	*RARE.hsp68LacZ* + DN-plasmid	E12.5→E15.5, P5	Impairment of CP entering	β-catenin	Choi et al., 2014
	(LOF)	E13.5→E16.5, P5	Altering the laminar fate		
		E14.5→E17.5, P5	Late-born neurons are affected		
		E15.5→E18.5, P5			

BP, bipolar; CP, cortical plate; DIV, day in vitro; E, embryonic day; GOF, gain of function; IUE, in utero electroporation; LOF, loss of function; MP, multipolar; Tmx, Tamoxifen.

### RP58 is a multifunctional regulator of cortical development

Recently, we found that the transcriptional repressor RP58, belonging to the POK/ZBTB proteins, which contain C-terminal zinc fingers and N-terminal BTB/POZ domains, has multiple roles in cortical development. RP58-gene- deficient mice die at birth and exhibit severe phenotypes associated with the proliferative state of NPCs and radial migration (Okado et al., 2009; Hirai et al., 2012; Ohtaka-Maruyama et al., 2013). A detailed analysis revealed that RP58 regulates cell cycle exit and neuronal migration by repressing its downstream targets. The RP58 gene was isolated originally from a screening for translin-associated molecules from a human spleen cDNA library (Aoki et al., 1998). It binds Dnmt3a and may be involved in transcriptional repression via chromatin remodeling (Fuks et al., 2001). Analyses of the spatial and temporal expression patterns during mouse brain development revealed that RP58 is weakly expressed in NPCs in the germinal zones of both the pallium and subpallium in the early developmental stage (Ohtaka-Maruyama et al., 2007). As development proceeds, RP58 is expressed strongly, via- Ngn2 activation in glutamatergic -neurons in the dorsal pallium. The peak expression of RP58 is at E15-16, when neurogenesis occurs most actively in the cortex by prominent promoter activity (Ohtaka-Maruyama et al., 2007, 2012). We demonstrated that RP58 enhances cell-cycle exit, resulting in neurogenesis via the transcriptional repression of Id1- 4 genes (Hirai et al., 2012). In RP58 deficient mice, cell cycle exit is impaired and the Pax 6- and PCNA-positive progenitor population is increased (Okado et al., 2009). Regarding the determination of laminar identity of differentiated neurons in RP58-deficient mouse cortex, neurons expressing the markers for layers II-V and SP neurons decreased remarkably, suggesting that RP58 plays a critical role in the maturation of cortical neurons (Okado et al., 2009). We also showed that RP58 controls MP-BP transition by regulating the Ngn2-Rnd2 pathway independent of its activity in the regulation of cell cycle exit (Ohtaka-Maruyama et al., 2013). Moreover, it has been revealed that RP58 represses Rnd2 transcription directly (Heng et al., 2015). These results suggested that RP58 enables transient expression of Ngn2 and restricts the Ngn2-Rnd2 signaling pathway both directly and indirectly. This regulation is essential for fine-tuning the Ngn2-Rnd2 signaling pathway to achieve proper radial migration. Furthermore, RP58 is involved in the regulation of neurite outgrowth (Ohtaka-Maruyama et al., 2013). Thus, RP58 is a multifunctional repressor for cortical development. Further functional analyses of RP58 will provide new insights into the molecular mechanisms of cortical development.

### Other transcriptional regulators involved in the multipolar-bipolar transition

FoxG1, a fate determinant factor as mentioned earlier, also plays an important role in radial migration (Miyoshi and Fishell, 2012; Table 1, Figure 3). Its transient downregulation in the IZ is critical for the MP-BP transition, suggesting that dose-dependent regulation of downstream targets is important for subsequent morphological and migration mode changes. *Laf4/Aff3*, a member of the AFF (AF4/FMR2) family, is known as a putative transcription factor and silencing of this gene is associated with neurodevelopmental disorders and intellectual disability (ID) (Steichen-Gersdorf et al., 2008; Metsu et al., 2014). However, its function in normal brain development is unclear. A recent study reported that *Laf4* is strongly expressed in the developing cortex and is required for the MP-BP transition via the transcriptional activation of *Mdga2*, a gene coding for a cell adhesion molecule (Moore et al., 2014; Table 1, Figure 3). Another intellectual disability-related gene, the X-linked intellectual disability protein PHF6 has also been reported to be associated with the PAF1 transcription elongation complex and to regulate MP-BP transition (Zhang et al., 2013a). The study showed that Neuroglycan C/Chondroitin sulfate proteoglycan 5(NGC/CSPG5), which is a potential schizophrenia-susceptibility gene, is a critical downstream target of PHF6 in this regulation (Table 1, Figure 3)

Prdm8 is a member of the proto-oncogene transcription family and has intrinsic histone methyltransferase activity (Hayashi et al., 2005; Eom et al., 2009). Prdm8 forms a repressor complex with Bhlhb5 and regulates neuronal circuit assembly through Cadherin-11 repression (Ross et al., 2012). Recently, it has been reported that Prdm8 regulates the MP-BP transition by maintaining the MP state and inducing morphological changes, and it controls genes including those encoding guidance molecules (Inoue et al., 2014; Table 1, Figure 3). Transcription factors Erm, Er81, and Pea3 belong to the Pea3 subfamily members of Ets (Pea3-Ets) and have also been reported to be involved in the regulation of the MP-BP transition (Table 1, Figure 3). It has been shown that the expression of these transcription factors is induced by FGF18-FGFR3 signaling in the developing cortex (Hasegawa et al., 2004). Knockdown of Erm, Er81, and Pea3 disrupt the entrance of migrating neurons into the CP, suggesting that Pea3-Ets transcription factors act as key mediators that interpret FGF signaling to confer proper migratory behavior on young MP neurons (Hasegawa et al., 2004). The molecular marker ER81 is expressed in a subset of pyramidal cells of layer V and also in NPCs at the mid- to-late stages of cortical development. Besides its association with the FGF signaling pathway, Pax6 has also been identified as an upstream activator of ER81, binding directly to the ER81 gene promoter (Tuoc and Stoykova, 2008).

In addition to transcription factors, it has recently been reported that microRNAs (miRNAs; miR) expressed in the developing cerebral wall are also involved in the regulation of radial migration (Clovis et al., 2012; Rago et al., 2014; Volvert et al., 2014; Table 1, Figure 3). miR369-3p, miR496, and miR543, all bind to the 3′-untranslated region of the N-cadherin (N-cad) transcript and regulate neurogenesis and neuronal migration by fine-tuning of N-cad levels (Rago et al., 2014). miR-9 and miR-132 target the 3′-untranslated region of the *Foxp2* transcript and regulate radial migration by controlling the *Foxp2* expression levels (Clovis et al., 2012). Meanwhile, the transcriptional repressor CoREST is also a miRNA target. miR-22 and miR-124 regulate proper expressional levels of CoREST, thereby regulating doublecortin transcription and promoting the MP-BP transition (Volvert et al., 2014). These lines of study suggest that minute transcriptional regulation of target genes including transcriptional factors and other proteins related to cortical development is critical for proper neuronal migration. Although RA signaling has long been known as an important regulator for neuronal development (Sockanathan et al., 2003; Fu et al., 2010), and activated RA receptor (RAR) is present in the developing dorsal and medial pallium (Luo et al., 2004), little is known about the function of RA in corticogenesis. RAR is a nuclear receptor that can act as a transcription factor. A recent study showed that inhibition of RAR function delays late-born neuron migration and leads to failure in maintaining their fate via β-catenin signaling (Choi et al., 2014). This suggests that RA signaling is critical for neuronal positioning as well as maintenance of their neuronal fate.

### Small GTP binding proteins in the multipolar-bipolar transition

Small GTP binding proteins (small GTPases, small G proteins), also known as the Ras superfamily, comprise more than 150 small G proteins that can be divided into five subfamilies: Ras, Rho, Rab, Arf, and Ran (Raimondi et al., 2010). They function as molecular switches in many cellular processes including cell proliferation, cytoskeletal organization, and cell migration. Although the Ras protein was originally recognized as an oncogene, it was recently revealed that small G proteins are indispensable for normal cellular functions including neuronal migration in the developing cerebral cortex (Kawauchi, 2011; Shah and Puschel, 2014). Among these, proteins belonging to the Rho, Ras, and Arf families or their regulator proteins have been reported to be involved in the MP-BP transition required for dynamic morphological changes (Figure 3). Rac1, regulator of maintenance of progenitor state is also involved in the regulation of the MP-BP transition. Functional repression of Rac1, its activators STEF/Tiam1, or its downstream molecule, c-Jun N-terminal kinase (JNK) resulted in defective MP-BP transitions of newborn cortical neurons (Kawauchi et al., 2003). This study revealed that Rac1 is essential for the MP-BP transition via regulating microtubule dynamics by activating JNK, followed by phosphorylation of MAP1B. P-Rex1, another activator of Rac (Rac-GEF), shows more restricted expression in neurons located at the lower part of the IZ of the mid-embryonic cortex, and participates in the regulation of radial neuronal migration via extracellular cues such as neurotrophins (Yoshizawa et al., 2005). Moreover, the Rac1-interacting scaffold protein POSH is required for the proper localization of activated Rac1 in the basal part of leading processes and regulates neuronal migration especially from the IZ into the CP (Yang et al., 2012a). These reports suggested that regulation of Rac1 is essential for proper MP-BP transition (Figure 3). α2-chimaerin is a Rac GTPase-activating protein (GAP) and was reported to be essential for neurite extension and axon pathfinding in the locomotor circuit and ocular system (Beg et al., 2007; Iwasato et al., 2007; Miyake et al., 2008). It has also been demonstrated that α2-chimaerin is essential for the MP-BP transition during radial migration (Ip et al., 2012). However, the function of this Rac-GAP protein is not dependent on its GAP activity, but rather via modulation of the activity of the microtubule-associated protein CRMP-2 (Ip et al., 2012; Figure 3). This result suggests that Rac regulator proteins have multiple functions mediated through different effectors. Rnd proteins are also important and unique Rho family members that lack intrinsic GTPase activity and are constitutively active. They regulate the actin cytoskeleton through inhibition of Rho-A signaling. As mentioned above with respect to Rnd2 function, fine-tuning of the Rnd2 level via transcriptional regulation is critical for the MP-BP transition of cortical migrating neurons (Heng et al., 2008, 2015; Alfano et al., 2011; Ohtaka-Maruyama et al., 2013; Figure 3). In contrast to Rnd2, Rnd3 regulates the early and late steps of radial migration, as described in Section Locomotion below. Some Ras family related proteins have been reported to be involved in MP-BP regulation, including Rap1 (Jossin and Cooper, 2011), RapGEF2 (Ye et al., 2014), and Dab2ip (Lee et al., 2012). Functional inhibition of Rap1 by IUE of Rap1GAP resulted in impairment of the MP-BP transition. Further analysis revealed that reelin-mediated activation of Rap1 in MP cells near the middle of the IZ increased the levels of cell- surface-localized N-cad, possibly by regulating vesicle trafficking of N-cad to ensure a proper morphological change to BP cells (Jossin and Cooper, 2011; Figure 3). The same signaling pathway, reelin-Rap1-N-cad, has also been reported to be essential for somal translocation of early born neurons and proper lamination of late-born neurons (Franco et al., 2011). The Rap1 activator RapGEF2 is expressed in the migrating neurons located in the upper IZ and CP. Short hairpin RNA (shRNA)-mediated knockdown of RacGEF2 prevents the MP-BP transition as well as N-cad recruitment to the cell membrane. RapGEF2 is activated by CDK5-dependent phosphorylation, suggesting that the CDK5-Rap1-N-cad signaling pathway is critical for exit from the MP phase (Ye et al., 2014). Furthermore, the Ras-GAP protein Dab2ip, which was initially identified as a tumor suppressor, is essential for the MP-BP transition *in vivo* and neurite outgrowth *in vitro* (Lee et al., 2012).

Periventricular heterotopia (PH) is a human cortical malformation disease associated with mutations in the *ArfGEF2* gene and the actin-binding protein Filamin A (FlnA) (Fox et al., 1998; Sheen et al., 2004). It has been shown that FlnA and its binding partner Filamin A-interacting protein (FILIP) are essential for the MP-BP transition by regulating the actin cytoskeleton (Nagano et al., 2002, 2004; Figure 3). Arf1 is a member of the Arf family and is involved in the regulation of vesicle trafficking, and *ArfGEF2* gene products (Big2 proteins) are GEFs for Arf1. *ArfGEF2* null mice develop PH and exhibit neuronal migration defects of the developing cortex (Zhang et al., 2012). Recently, it was revealed that Big2 and FlnA interact directly and regulate neuronal migration and cell adhesion through modulation of Arf1 activity and localization of Big2 to the cell membrane from the Golgi (Zhang et al., 2013b). Arf6 is another Arf family member regulating the MP-BP transition. TBC1 domain family member 24 (TBC1D24) is an Arf6-interacting protein, and mutations in the TBC1D24 gene are associated with cortical malformation, intellectual disability, and epilepsy (Corbett et al., 2010; Falace et al., 2010). Recent finding revealed that TBC1D24 is essential for the MP-BP transition and dendritic arborization through Arf-GAP activity, which prevents Arf6 activation (Falace et al., 2014; Figure 3).

As described above, small G-proteins involved in the regulations of membrane trafficking, cytoskeletal organization and cell adhesion play critical roles in dynamic morphological changes during the MP-BP transition during radial migration of neuroblasts. It is undeniable that many causative genes of neurodevelopmental diseases belong to small G-protein family.

### Regulation of microtubule (MT) dynamics in the multipolar-bipolar transition

*Lissencephaly-1 (LIS1)* is the first identified gene responsible for type I lissencephaly (Reiner et al., 1993). Since then, many lines of evidence have revealed that LIS1 regulates neuronal migration in a dose-dependent manner (Youn et al., 2009; for a review, see Reiner and Sapir, 2013). LIS1 is an MT or microtubule organizing center (MTOC)-associated protein that forms a protein complex with NDE1/NDEL1 and cytoplasmic dynein (Feng et al., 2000; Sasaki et al., 2000). LIS1 is essential for INM, axon extension, and the MP-BP transition by functioning with NDE1/NDEL1 (Tsai et al., 2005; Youn et al., 2009). For the nuclear migration of radially migrating cells, coordinated coupling between translocation of the centrosome and subsequent nuclear movement via dynamic MT organization is essential, and disruption of this coordination leads to failure to enter the CP. In this context, using the MADM system, the distinct and cell-autonomous functions of LIS1 and NDEL1 in neuronal migration have been revealed. LIS1 regulates this step in a dose-dependent manner, whereas NDEL1 is indispensable for entering the CP (Hippenmeyer et al., 2010). It has been reported that NDEL1 also forms a protein complex with DISC1 and Dixdc1 to regulate radial migration; Cdk5 phosphorylation of Dixdc1 is essential for this regulation (Singh et al., 2010; Figure 3).

The centrosome is composed of two orthogonally arranged centrioles surrounded by proteinaceous materials called pericentriolar materials called PCM, which contain many proteins required for MTOC activity, including γ-tubulin, PCM-1, pericentrin and ninein (Dammermann and Merdes, 2002; for a review, see Bornens and Gonczy, 2014). Centrosome positioning has been shown to be important for neuronal polarity establishment (de Anda et al., 2010). A recent time-lapse imaging study of centrosome positioning during the MP-BP transition revealed that centrosomes exhibit the motion feature that targets the basal part of the dominant growing process in MP-migrating neurons (Sakakibara et al., 2014). Another study revealed that the centrosomal protein SDCCAG8 regulates the MP-BP transition through interaction with PCM1 and centrosomal recruitment of PCM (Insolera et al., 2014; Figure 3). Knockdown of *SDCCAG8* impairs coordinated coupling of movements of the centrosome and nucleus (Insolera et al., 2014). Mutations of *SDCCAG8* are known to be associated with the human diseases such as nephronophthisis (Otto et al., 2010) and Bardet-Biedl syndrome (BBS) (Schaefer et al., 2011) a rare autosomal recessive ciliopathy with various symptoms, including renal and retinal abnormalities (Forsythe and Beales, 2013). Patients with mutations in *SDCCAG8* often exhibit neurodevelopmental disorders, including mental retardation, cognitive impairment, and seizures, suggesting a roles for SDCCAG8 in brain development (Otto et al., 2010). Taking this into account, neuronal migration defects occurring by knockdown of this centrosomal protein in the developing mouse cortex could contribute to be explained by the neurodevelopmental symptoms of Bardet-Biedle syndrome.

### Receptors and other membrane proteins involved in the MP-BP transition

Membrane proteins located on the cell surface are important for translating extracellular signals into intracellular signal transduction. Neuroblasts should receive signals from the extracellular space to change their morphology and to acquire their neuronal properties. Actually, a proportion of the reported genes regulating the MP-BP transition are localized in the cell membrane and play critical roles in this regulation including receptors, gap junction protein, and transmembrane glycoprotein.

Unc5D is known as a receptor for the guidance molecule Netrin, and is expressed in MP cells in the SVZ during cortical development (Sasaki et al., 2008). *Svet1* RNA (Tarabykin et al., 2001), which is a known SVZ marker, has been shown to be derived from an intronic region of the *Unc5d* gene locus (Sasaki et al., 2008). Yamagishi et al. (2011) reported that fibronectin and leucine-rich transmembrane protein-2 (FLRT2) are novel repulsive guidance molecules for Unc5D receptors in radial neuronal migration. They further found that the extracellular domains (ECDs) of FLRT2 proteins are shed by proteolytic cleavage and soluble FLRT2 ECDs regulate MP cells in entering the CP. A recent analysis of the crystal structure of the FLRT2-Unc5D-complex confirmed the ligand-receptor-binding site and three-dimensional structure (Seiradake et al., 2014; Figure 3). As mentioned previously, downregulation of the transcriptional repressor FoxG1 at the beginning of the MP cell phase contributes to induction of Unc5D expression (Miyoshi and Fishell, 2012). Whereas, for reduction of Unc5D expression, PRDM8 has been suggested to contribute to this regulation of the MP-BP transition (Inoue et al., 2014).

Connexin 43(Cx43) is a gap junction protein that assembles a hemichannel of large- diameter channels in gap junctions. It has been reported that Cx43 is necessary for neuronal migration, especially the MP-BP transition independent of its channel-forming activity (Fushiki et al., 2003; Elias et al., 2007; Figure 3). Cx43 plays an important role in the adhesion of migrating neurons to RG fibers to stabilize their leading processes (Elias et al., 2007). A recent study revealed that Cx43 controls the MP phase via p27^kip1^ upregulation; this Cx43-p27^kip1^ signaling is mainly dependent on the adhesive function of Cx43, although there is an auxiliary role of the Cx43 C-terminus (Cina et al., 2009; Liu et al., 2012), which can interact with variety of proteins related to the cytoskeleton (Herve et al., 2012). These lines of evidence suggest that the membrane proteins of migrating MP neurons play crucial roles in translating the extrinsic signal into intracellular information required for cytoskeletal reorganization of leading process formation and stabilization.

Amyloid-β precursor protein (APP) is a type I transmembrane glycoprotein, and its proteolysis product Aβ accumulates in neurons in Alzheimer's disease. However, the cellular function of APP in normal neuronal development remains unknown. Young-Pearse et al. (2007) found that knockdown of APP using IUE resulted in impairment of entering the CP during radial migration of the developing cortex. In this study, the authors also revealed that full-length APP is required for this function, which is regulated by the downstream adaptor protein Disabled-1 (Dab1). Recently, a secreted glycoprotein pancortin was identified as an extracellular binding partner of APP (Rice et al., 2012; Figure 3). Pancortin is expressed at high levels in the developing and mature mouse cortex, and the pancortin gene encodes four isoforms. Rice et al. (2012) further revealed that although all four isoforms of pancortin biochemically interact with APP, each isoform regulates the MP-BP transition in a different manner together with APP.

Recently, it was reported that serotonin 6 receptor (5-HT6R), a G protein-coupled receptor (GPCR), is critical for the MP-BP transition and locomotion (Jacobshagen et al., 2014). This function was also found to depend on CDK5 by binding to the intracellular region of 5-HT6R, but was independent of serotonin activation. This suggests that 5-HT6R is an upstream membrane regulator for CDK5 function in neuronal migration (Jacobshagen et al., 2014; Figure 3).

### Protein kinases involved in the multipolar-bipolar transition

Protein kinases are a key class of regulatory proteins for many cellular functions. Protein phosphorylation is broadly known as a molecular switch for downstream pathways. It has been reported that protein kinases play a critical roles in the MP-BP transition.

CDK5 is a serine/threonine kinase that plays crucial roles in brain development. CDK5 knockdown in migrating neurons leads to impairment of leading process formation and the MP-BP transition (Kawauchi et al., 2006; Ohshima et al., 2007), suggesting its critical roles in regulating MT or actin cytoskeletal organization. Accumulating evidence has uncovered the molecular pathways and identified its downstream substrates, including the CDK inhibitor p27^kip1^ (Kawauchi et al., 2006), the kinase Mst3 (Tang et al., 2014), the scaffold protein axin (Fang et al., 2013), the actin-binding protein drebrin (Tanabe et al., 2014), and MT-associated proteins, including DCX (Tanaka et al., 2004), FAK (Xie et al., 2003), NDEL1 (Niethammer et al., 2000; Sasaki et al., 2000), and CRMP2 (Uchida et al., 2005). CDK5 functions as a master kinase for neural development (Figure 3). For details of CDK5 pathways in neuronal migration, please see Ohshima's (2014) review in this Research Topic issue.

LKB1, originally identified as an ortholog of the Par4 serine/threonine kinase of *Caenorhabditis. elegans*, has been reported to be involved in axon specification *in vivo* (Asada et al., 2007; Barnes et al., 2007; Shelly et al., 2007). A pseudokinase Ste20-related kinase adaptor α (STRADα̣ can stabilize LKB1 (Veleva-Rotse et al., 2014), and LKB1 is activated by protein kinase A-dependent local phosphorylation on S431 in the trailing processes of newborn migrating neurons, followed by activation of the downstream kinases SAD-A and SAD-B, both of which are known to be essential for axon formation by phosphorylating Tau-1 (Kishi et al., 2005; Barnes et al., 2007). *STRAD*α has been identified as the gene responsible for the autosomal recessive neurodevelopmental disorder polyhydramnios, megalencephaly, and symptom epilepsy syndrome (PMSE), which is characterized by macrocephaly, craniofacial dismorphism, hypotonica, cognitive disability, and intrac epilepsy (Puffenberger et al., 2007). STRADα binds LKB1 together with the scaffold protein MO25 and facilitates nuclear export of LKB1 to the cytoplasm (Boudeau et al., 2003; Zeqiraj et al., 2009). It has been reported that human PMSE cortex exhibited abnormal nuclear localization of LKB1 (Orlova et al., 2010). The STRAD/ LKB1 complex inhibits mammalian target of rapamycin (mTOR) signaling via AMP-activated kinase (AMPK) and tuberous sclerosis complex 1 and 2 (TSC1, TSC2) (Inoki et al., 2003; Corradetti et al., 2004; Lizcano et al., 2004). Knockdown of STRADα in mouse NPCs *in vitro* resulted in aberrant mTORC1 activation and abnormal nuclear localization of LKB1. Moreover, Knockdown of STRADα *in vivo* also leads to aberrant mTORC1 activation and impairment of radial neuronal migration (Orlova et al., 2010). Acute inactivation of the STE family serine/threonine kinase Stk25, which is another binding partner of STRADα, causes impairment of the MP-BP transition. These results suggests that STRADα-Stk25-LKB1- mTORC1 signaling, may regulate radial neuronal migration in addition to its role in polarity formation (Matsuki et al., 2010, 2013) and also suggests that hyperactivation of mTORC1 signaling by STRADα gene mutation affect the cortical development at an early stage in human PMSE (Figure 3). Through sh-RNA-mediated knockdown of LKB1 by IUE, it has been revealed that LKB1 actually regulates the transition of MP to BP in addition to its role in axon formation (Asada et al., 2007). The authors further revealed that this migration defect is correlated with a defect in centrosomal movement, and that LKB1 mediated inactivation of GSK3β by Ser9 phosphorylation at the leading process tip to stabilize the MT plus-end-binding protein APC for proper forward movement of centrosomes (Asada et al., 2007; Asada and Sanada, 2010). By contrast, other studies did not observe any migration defect by silencing LKB1 (Barnes et al., 2007; Shelly et al., 2007). Although this discrepancy remains to be resolved, all of these experiments of LKB1 knockdown or knockout were performed at different time periods and in different conditions. Moreover, overexpression of LKB1 exhibits a migration defect (Shelly et al., 2007), suggesting the possibility that the requirement of LKB1 for neuronal migration may critically depend on its protein level.

MAP/microtubule affinity- regulating kinase 2 (MARK2/Par-1) is another polarity kinase involved in the regulation of centrosome dynamics (Sapir et al., 2008a,b). MARK2 regulates MT dynamics by phosphorylating the MAPs tau, MAP2/4, and Dcx (Drewes et al., 1997; Schaar et al., 2004). Sapir et al. (2008a), have revealed that reduction of MARK2 in migrating neurons impairs centrosomal movement by centrosome-nucleus decoupling, which results in a defect in the morphological change from MP to BP (Sapir et al., 2008a). They also showed that tight regulation of MARK2 activity, followed by phosphorylation of Dcx and destabilization of MTs is essential for proper neuronal migration (Sapir et al., 2008b; Figure 3).

Mammalian Ste2-like kinase 3 (Mst3) is known to regulate axogenesis of dissociated cultured neurons (Irwin et al., 2006; Lorber et al., 2009). A recent study revealed that the novel signal pathway of CDK5-Mst3-RhoA is involved in regulating the MP-BP transition (Tang et al., 2014). Knockdown of Mst3 expression by delivery of shRNA constructs using IUE results in a migration defect of MP cells to convert into BP cells and enter the CP. This function of Mst3 is dependent on S79 phosphorylation by CDK5 as an upstream regulator, and modulation of RhoA activity for regulating the actin cytoskeleton as a downstream effector (Tang et al., 2014; Figure 3).

## Locomotion

**Figure 4:**
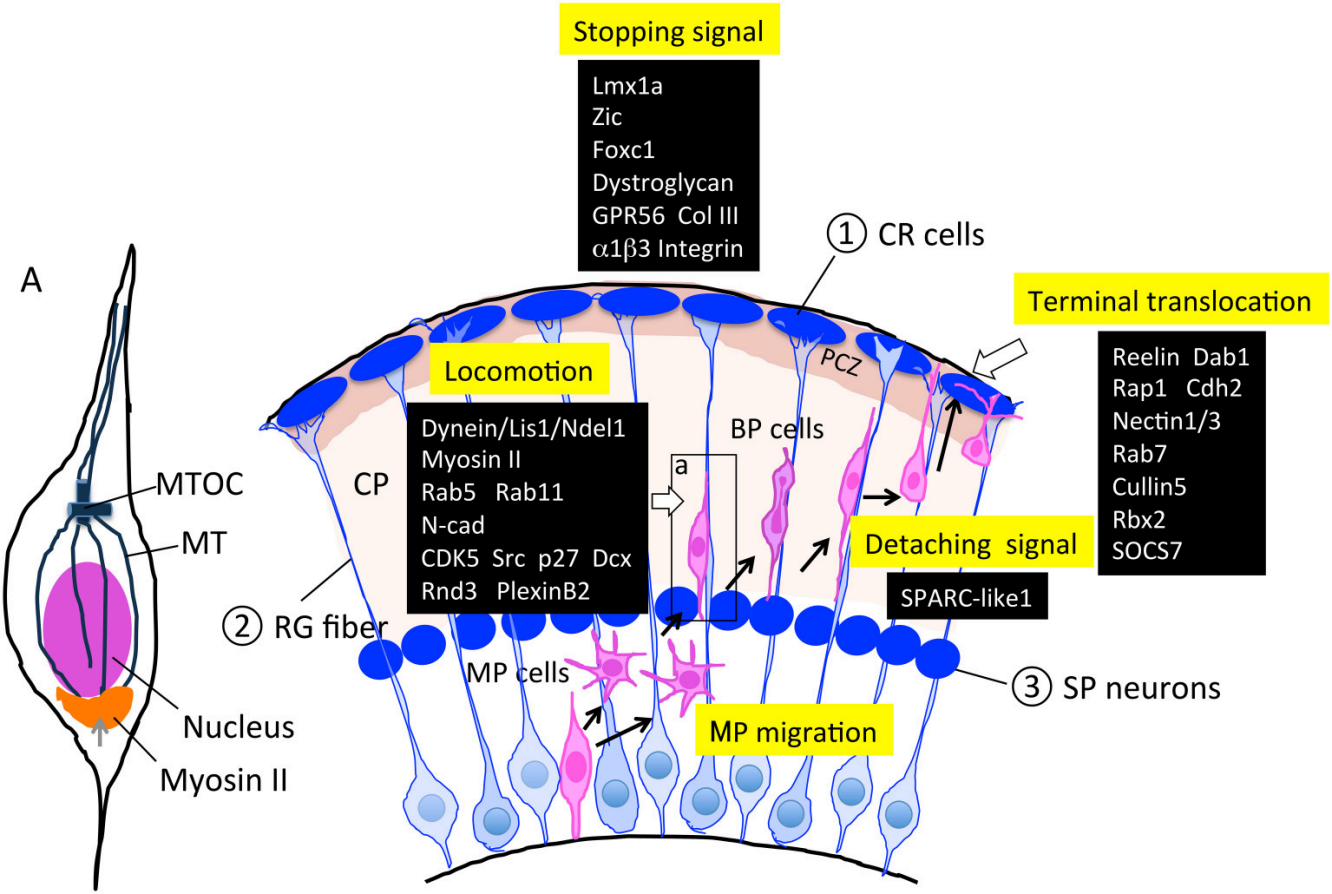
**Molecules and structures involved in the locomotion and termination of radial migration**. Cajal-Retzius (CR) cells, radial glial (RG) fibers, and subplate (SP) neurons are transient cell structures that mostly disappear after birth. As described in Sections Locomotion and Termination of Radial Migration, various factors are involved in this regulation. PCZ, primitive cortical zone (Sekine et al., 2011) A; an enlarged neuron during locomotion. The microtubule organizing center (MTOC) extends the MT toward the tip of the leading process and toward the trailing process that forms a cage-like structure surrounding the nucleus. Myosin II has been shown to localize to the peri-nuclear region (bottom portion), and contributes to moving the nucleus up to the MTOC during locomotion. BP, bipolar; CP, cortical plate; CR, Cajal-Retzius; MP, multipolar; MT, microtubule; MTOC, microtubule organizing center; RG, radial glia; SP, subplate.

After converting to a BP shape, neurons execute RG-guided locomotion toward the pial surface (Figure 4). This migrating movement is completely different from MP migration, suggesting that a distinct set of genes are upregulated or downregulated, and reorganization of signaling pathways may occur using the same protein members in locomoting cells.

In the locomotion step, coupled movement of the centrosome and nucleus by cytoskeletal coordination is essential for nuclear translocation, and endocytosis and neuronal adhesion are involved in the forward movement of the cell body (Kawauchi et al., 2010). The centrosomes (also called MTOCs) are located in the proximal part of the leading processes, and MTs project from the MTOC anteriorly toward the leading process tip and posteriorly toward the nucleus. MTs that surround the nucleus form a fork-like structure that may contribute to pulling the nucleus forward (Xie et al., 2003). The dynein/LIS1/NDEL1 complex plays an essential role in this regulation (Tsai et al., 2007), as described above. Myosin II is another motor protein that produces force for nuclear movement (Figure 4A; Schaar and McConnell, 2005). Activated myosin II localized at the rear of the cell generates a pushing force on the nucleus, which is normally coordinated with a centrosome-MT-dynein-mediated pulling force of the nucleus, suggesting that cytoskeletal coordination is essential for proper nucleokinesis during locomotion (Schaar and McConnell, 2005).

With respect to the molecular mechanisms for locomotion, Kawauchi et al. (2010) revealed that Rab-dependent membrane trafficking pathway is essential for locomotion as well as terminal translocation. Using the IUE technique, they further clarified the *in vivo* roles of endocytotic pathways in neuronal migration. In particular, they revealed that the Rab5-dependent endocytosis and Rab11-dependent recycling pathways are essential for the locomotion step, and that N-cad may be one of the major target molecules of this pathway. Whereas, Rab7 knockdown affected terminal translocation, suggesting that Rab7-dependent lysosomal degradation pathways including N-cad as one of these substrates, contribute to the final phase of radial migration.

Using *ex vivo* chemical inhibitor screening and time-lapse imaging of cultured slices of the electroporated embryonic cortex, Nishimura et al. (2010) found that roscovitine and PP2, inhibitors of CDK5 and Src-family kinases, respectively, reduce locomotion speed, suggesting the involvement of the activities of these kinases in the locomotion mode. During locomotion, migrating neurons continue to repeat the morphological change; following extension of the leading process, dilation is formed at the forward part of the nucleus, thereby moving the centrosome into the dilation, which is followed by nuclear elongation and translocation. Recently, Nishimura et al. (2014) reported that CDK5 and its downstream substrates Dcx and p27^kip1^ regulate these cytoplasmic dilation and nuclear translocation steps. Moreover, dynamin and Rab-5-dependent regulation of endocytosis regulated by CDK5 is involved in this pathway (Nishimura et al., 2014).

Rnd3, another member of the Rnd protein family, has been reported to be involved in the regulation of neuronal migration via a mechanism distinct from that of Rnd2 (Pacary et al., 2011). In contrast to Rnd2, in which transcription is directly by Ngn2, Rnd3 is a direct target of Ascl1. Although both Rnd2 and Rnd3 inhibit Rho-A activity, compared with Rnd2, which is localized in the soma and regulates the MP-BP transition, Rnd3 is associated with the plasma membrane, where it inhibits and regulates locomotion by repressing F-actin polymerization. It was reported recently that the semaphorin receptor, plexin B2 interacts with Rnd3 and antagonizes the binding of Rnd3 to the Rho-A suppressor p190 RhoGAP, whereas, plexinB2 activates RhoA through recruiting Rho-GEFs (Azzarelli et al., 2014). This suggests that antagonizing regulation by an extrinsic semaphorin signal and an intrinsic Ascl1 signal is critical for maintaining appropriate RhoA activity required for locomotion. In contrast to these studies, another study using *Emx::Cre/RhoA fl/fl* (cKO) mice showed that RhoA activity is dispensable for migrating neurons, but is required for proper formation of the RG scaffold (Cappello et al., 2012). This discrepancy could be explained by compensative activities for Rho-A in the knockout neurons, but the mechanism is still up for debate.

In summary, some proteins such as CDK5, Lis1, Ndel, and N-cad play important functions in both steps of the MP-BP transition and locomotion. Others, like Rnd proteins (Rnd2 and Rnd3), as mentioned above, have distinct roles in each step although they belong to the same protein family. This suggests that a distinct set of genes participate in regulation of locomotion to fulfill the switching of migration mode during cortical evolution, as we discuss later.

## Termination of radial migration

Finally, when migrating neurons arrive at their final destinations, they terminate migration and shift to terminal translocation and maturation by extending their axons and dendrites. This termination step should be executed by at least three distinct behaviors of locomoting neurons: locomotion termination, detachment from the RG fiber, and anchoring to the MZ for terminal translocation (Figure 4).

Impairment of the stopping signal results in neuronal over-migration into the meninges, leading to neocortical dysplasia resembling cobblestone (type II) lissencephaly with a defect in basement membrane (BM) integrity. This includes over-migration of the early born neuronal population as well as late-born locomoting neurons. Many factors have been reported to be involved in this over-migration defect, including the LIM homeobox gene *Lmx1a* (Costa et al., 2001), the zinc finger transcription factor Zic (Inoue et al., 2008), the forkhead box transcription factor Foxc1 (Hecht et al., 2010), dystroglycan protein (Myshrall et al., 2012), and the GPCR GPR56 (Singer et al., 2013). The brains of mice with mutations in any of these exhibit defects in the interaction between pial basement membrane (PBM) and RG processes and PBM integrity. These defects bring about over-migration of neuroblasts into the meningeal space, followed by disorganization of the laminar structure. Among these factors, the molecular mechanism of the defects is most well studied with respect to GPR56. The gene encoding this orphan GPCR was originally identified to be responsible for the human recessively inherited genetic disorder bilateral frontoparietal polymicroglia (BFPP), which is a cobblestone-like brain malformation (Piao et al., 2004; Bahi-Buisson et al., 2010). Luo et al. (2011), identified collagen III as the ligand for GPR56, and the ligand binding triggers RhoA activation via coupling to Gα_12∕13_. Mutations in the ligand-binding domain of GPR56 have been found in BFPP patients, suggesting the importance of Collagen III (ColIII)-GPR56 signaling for PBM integrity to prevent neuronal over-migration of preplate neurons. The same research group found that α3β1 integrin functions together with GPR56 to produce a proper stopping signal (Jeong et al., 2013; Figure 4).

SPARC-like 1 (SC1), a member of the SPARC family of ECM proteins, is involved in detaching locomoting neurons from the RG fibers, and is expressed at the top and bottom of the RG cell surface. SC1 was identified via antigen screening for radial glial immunoreactive monoclonal antibodies. SC1 possesses an anti-adhesive activity that may contribute to detaching the locomoting neurons from the RG fibers at their final step of locomotion. The absence of SC1 results in defective termination of locomotion and the final positioning of neurons, suggesting that anti-adhesive signaling at the termination phase is essential (Gongidi et al., 2004; Figure 4).

Reelin plays an essential role in terminal translocation. Reelin is an extracellular protein secreted from CR cells in the MZ, and extensive analyses have revealed the molecular mechanisms of reelin signaling in neuronal migration (for a review, please see Sekine et al., 2014). Nakajima's group has found a novel thin layer at the outermost region of the mouse cortical plate that is histologically distinct and characterized by densely packed and NeuN-negative immature neurons called the primitive cortical zone (PCZ) (Sekine et al., 2011). This group also revealed that locomoting neurons must enter the PCZ in order to switch their migration mode to terminal translocation. This step is dependent on the reelin-Dab1 signaling pathway and is critical for completion of inside-out lamination at the final stage of radial migration (Kubo et al., 2010; Sekine et al., 2011).

Muller's group has revealed that the reelin-Dab1-Rap1-cadherin signaling pathway is essential for terminal translocation as well as for inside-out lamination (Franco et al., 2011). They also found that adhesion molecules Nectin1 and 3 are indispensable for terminal translocation via Rap1-mediated stabilization of cell surface Cdh2 (Gil-Sanz et al., 2013).

Furthermore, Cooper and colleagues revealed that the regulation of Dab1 degradation by the ubiquitin-proteasome system is critical for producing the stopping signal of migrating neurons (Arnaud et al., 2003). They further uncovered the molecular mechanism for this regulation, in which the E3 ubiquitin ligase complex, including Cullin 5, Rbx2, and their adaptor protein SOCS7, are critical for inhibiting over-migration (Simo et al., 2010; Simo and Cooper, 2013; Figure 4).

## Cortical evolution and radial migration

So far, we have examined each step in radial migration of the developing cortex from the viewpoint of the molecular pathways involved. Now we focus on neuronal migration from an evolutionary perspective.

The six-layered laminar structure of the neocortex is unique to mammals (Nieuwenhuys, 1994). Pyramidal neurons are born sequentially in the VZ and migrate toward the pial surface. In this process, late-born neurons pass early born neurons and form a neuronal layer on top of the older layer in an inside-out manner. In contrast, non-mammalian, amniote brains, such as those of reptiles and birds, do not have this inside-out type layer structure (Northcutt and Kaas, 1995; Molnar et al., 2006; Nomura et al., 2009, 2013). The reptilian cortex is organized in a three-layered structure, but not in an inside-out manner, and the avian cortex contains a nucleus structure instead of a laminar structure. This evolutionary transition raises the question of what is the advantage of the mammalian-specific laminae of the neocortex? A simple analogy that can be used to answer this question is that of a bookshelf. The volume of information and accessibility of the content is markedly different between a highly organized six-shelf bookcase and disorganized stacks of books on the floor in the same space. One can easily find a book of interest on the organized bookshelf much faster than from the stacks of books. For corticogenesis, axon pathfinding of organized neurons in the six-layer structure may easier to reach the targets and more neuronal connections could be formed compared with neurons in a nuclear structure. Among the ancestral amniotes, primitive mammals acquired the layered structure of the cerebral cortex during evolution. Moreover, primate brains evolved remarkable expansion of the neocortex area based on this structure (Molnar et al., 2006; Lui et al., 2011). During development of the mammalian neocortex, there are at least three transient cell structures that mainly appear in the developing stage to assist with completion of radial neuronal migration: CR cells, RG fibers, and SP neurons (Voigt, 1989; Kostovic and Rakic, 1990; Misson et al., 1991; Arias et al., 2002; Kirischuk et al., 2014; Figure 4). These cell structures contribute to execution of mammalian-type neuronal migration (Xie et al., 2002; Nomura et al., 2008); newborn neurons convert from MP cells to BP cells and migrate toward the pial surface in locomotion mode, followed by terminal translocation and maturation. Furthermore, OSVZ progenitor cells (bRG), a third population of neural progenitors, have been identified in the mammalian developing cortex (Fietz et al., 2010; Hansen et al., 2010; Shitamukai et al., 2011; Wang et al., 2011b). This progenitor population is observed more frequently in the primate cortex and is thought to contribute to cortical expansion and gyrification in the process of neocortical evolution. The bRG cells undergo mitotic somal translocation (MST), and the cell soma rapidly ascends along the basal fiber before cytokinesis (Hansen et al., 2010; Wang et al., 2011b). Although the specific migration mode of somal translocation in the non-mammalian developing cortex has not yet been reported, the somal translocation-type migration mode, including terminal translocation and MST, might have been acquired during the neocortical evolution of mammals as well as the locomotion mode. It is intriguing to examine the migration types of the non-mammalian amniote embryonic cortex using time-lapse imaging. Taken together, the switching of migration modes during radial migration of newborn pyramidal neurons may have been essential for evolution of the mammalian-type neocortex.

### The subplate layer and switching of migration mode

**Figure 5:**
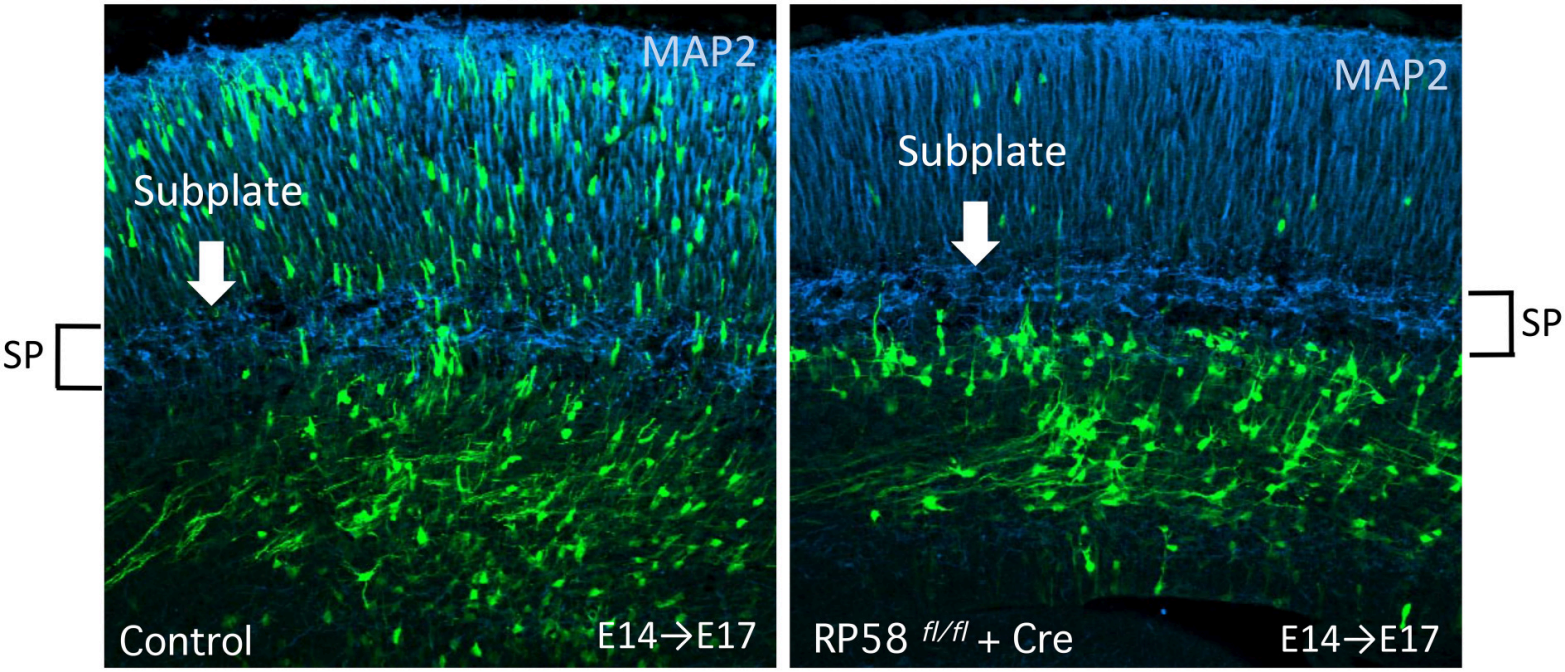
**Acute deletion of RP58 results in MP-BP transition**. Green fluorescent protein (GFP)-positive cells labeled by IUE at E14 were analyzed at E17. The figure shows Cre-mediated RP58 knockout cells are stacked just under the SP that can be recognized distinctly by MAP2 immunostaining. (The data is Figure 3D from Ohtaka-Maruyama et al., 2013) E, embryonic day; IUE, *in utero* electroporation; SP, subplate.

**Figure 6:**
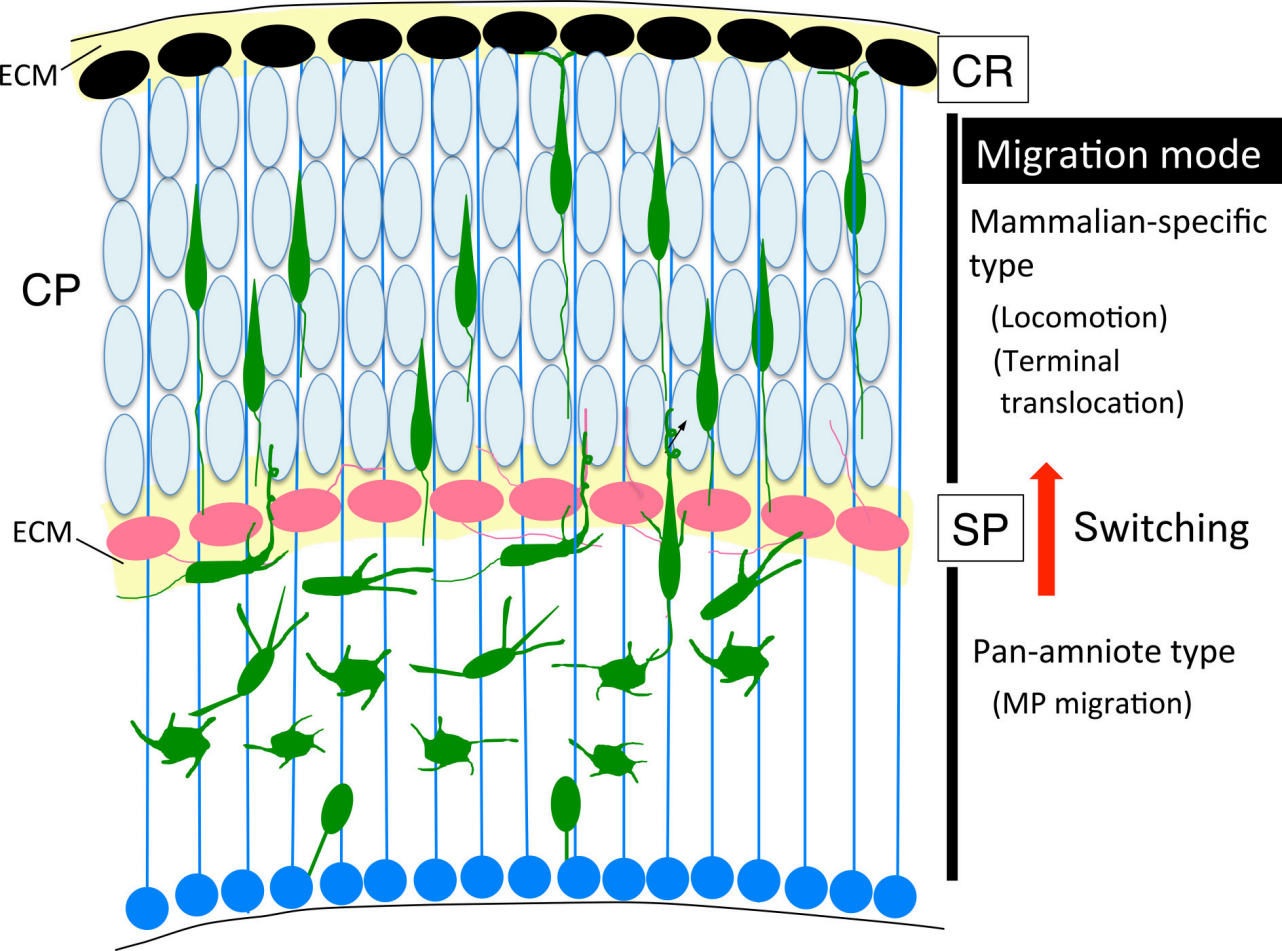
**Cortical evolution and neuronal migration**. The switching of migration modes, from MP migration (pan-amniote type) to locomotion (mammalian-type) might occur at the SP layer during cortical evolution. BP, bipolar; CP, cortical plate; CR, Cajal-Retzius; ECM, extracellular matrix; MP, multipolar; SP, subplate.

Recently, we revealed that migrating neurons that lack RP58 can migrate to the subplate (SP) layer, but are stacked just below the SP (Figure 5; Ohtaka-Maruyama et al., 2013). However, we noticed that knockdown or knockout of the expression of many other genes in migrating neurons resulted in a remarkably similar phenotype, including CDK5, the chondroitin sulfate modifying enzyme GalNAc4S-6ST, the Rac inhibitor α2-chimaerin, the Rap1 activator RapGEF2, and others (Ohshima et al., 2007; Ishii and Maeda, 2008; Ip et al., 2012; Ye et al., 2014). This suggests that the SP layer acts as a specific barrier for migrating neurons to cross over. As described in Section Multipolar Cell to Bipolar Cell Transition, it has been reported that various molecular pathways are involved in regulation of the MP-BP transition. When we carefully observed the cortical sections prepared from the electroporated cortex, we could detect that the SP layer resides in a boundary of the MP-BP transition. MP cells or the transit type to BP cells with multiple neurites were observed below the SP, whereas migrating cells that crossed over the SP possessed thick leading processes and migrate in locomotion mode. Accordingly, a cue to start the signaling pathway of the MP-BP transition may be received by the MP migrating cells when they reach the SP layer. It is conceivable that impairment of this cue or any of the downstream signals would lead to stacking of the MP cells just below the SP layer owing to failure in the transition to BP cells. We hypothesized that this signaling pathway at the SP played a critical role in the evolution of migration mode from the avian-type MP migration to mammalian-type locomotion and terminal translocation (Figure 6). The SP layer of the embryonic cortex is rich in ECM, including fibronectin, proteoglycan (CSPG; phosphacan, versican, neurocan, aggrecan), and collagen (collagen11a1) (Sheppard et al., 1991; Maeda et al., 1995; Meyer-Puttlitz et al., 1996; Popp et al., 2003; Hoerder-Suabedissen et al., 2013). Therefore, many signaling molecules could be held in the SP layer during corticogenesis. By elucidating the functional roles of the SP layer in radial neuronal migration, it is anticipated that we can start to resolve the question of how the mammalian neocortex evolved to the present six-layered inside-out structure.

## Perspective

Our review of recent progress in understanding the molecular pathways involved in radial neuronal migration of glutamatergic neurons during corticogenesis revealed that proper regulation of the various signaling pathways operating inside or outside of newborn neurons (neuroblasts) is critical for each step of neuronal migration. In general, accurate control of developmental timing for gene expression, translational control, and modification of protein activity is required for embryonic morphogenesis. To understand the regulatory mechanisms, it is important to determine how extrinsic signaling cues translate into intracellular signals. Newborn cortical neurons execute a dynamic morphological change from MP cells to BP cells. During this step, axons are first determined, followed by formation of thick leading processes, before making the transition to locomotion mode. Although the many factors involved in regulation of polarity formation *in vitro* were identified for axon determination, more signaling pathways are required for leading process formation, suggesting the importance of *in vivo* signaling from the extracellular environment.

So far, explorations of gene function have been most commonly performed using knockdown or overexpression experiments, by delivering the DNA constructs with the IUE technique or via genetic modification of the gene locus to establish conventional and conditional knockout mice. However, to achieve more comprehensive understanding of these complex processes beyond determination of individual gene function, we could apply these conventional techniques in combination with more advanced techniques. For example, the MADM system or CLoNe (Garcia-Moreno et al., 2014) allows for clonal analysis, and double electroporation with two different colors in different stages is useful for analyzing the interaction between two cell populations. Moreover, time-lapse imaging of cultured slices of the cortex using calcium indicator-encoding plasmids or a channel rhodopsin system could reveal *in vivo* neuronal activities and the effects of activity manipulation. To investigate protein-protein interactions, or protease activity on the ECM, the use of fluorescent probes such as the proximity ligation assay and protease imaging could be effective techniques.

In conclusion, by using these imaging techniques in combination with conventional genetic manipulation, we could advance our understanding of the molecular mechanisms underlying neuronal migration and brain development *in vivo*. Finally, we would like to emphasize that it is critical to consider an evolutionary perspective in order to understand brain development as a whole system.

### Conflict of interest statement

The authors declare that the research was conducted in the absence of any commercial or financial relationships that could be construed as a potential conflict of interest.

## Abbreviations

aIP: apical intermediate progenitor
AMPK: AMP-activated kinase
AP: apical progenitors
APP: amyloid-b precursor protein
aRG: apical radial glia
ASD: autism spectrum disorder
bIP: basal intermediate progenitor
BM: basement membrane
BMP: bone morphogenetic protein
BP: basal progenitors or bipolar
bRG: basal radial glia
CDK: cyclin dependent kinase
CNS: central nervous system
CP: cortical plate
CR: Cajal Retzius
CSF-1: colony stimulating factor-1
E: embryonic day
ECD: extracellular domain
FGF: fibroblast growth factor
FGFR: fibroblast growth factor receptor
GAP: GTPase-activating protein
GEF: guanine nucleotide exchange factor
GPCR: G-protein coupled receptor
IL: interleikin
INM: interkinetic nuclear migration
IP: intermediate progenitor
IUE: *in utero* electroporation
IZ: intermediate zone
JNK: c-Jun N-terminal kinase
MADM: mosaic analysis with double markers
MAZ: multipolar cell accumulation zone
miRNA: microRNA
MST: mitotic somal translocation
MTOC: microtubule organizing center
MP: multipolar
MT: microtubule
mTOR: mammalian target of rapamycin
N-cad: N-cadherin
NPC: neural progenitor cell
OSVZ: outer subventricular zone
PBM: pial basement membrane
PcG: polycomb group
PCM: pericentriolar material
PH: periventricular heterotopia
PMSE: polyhydramnios, megalencephaly and symptom epilepsy syndrome
PSB: pallial-subpallial boundary
RA: retinoic acid
RAR: retinoic acid receptor
REP: rapidly exiting population
RG: radial glia
RGC: radial glial cell
SEP: slowly exiting population
shRNA: short hairpin RNA
SP: subplate
SVZ: subventricular zone
TACC: transforming acidic coiled coil proteins
TSC: tuberous sclerosis complex
VZ: ventricular zone
WT: wild-type.
