# Corrigendum: Molecular Pathways Underlying Projection Neuron Production and Migration during Cerebral Cortical Development

**DOI:** 10.3389/fnins.2016.00160

**Published:** 2016-04-12

**Authors:** Chiaki Ohtaka-Maruyama, Haruo Okado

**Affiliations:** ^1^Neural Network Project, Department of Brain Development and Neural Degeneration, Tokyo Metropolitan Institute of Medical ScienceTokyo, Japan; ^2^Neural Development Project, Department of Brain Development and Neural Degeneration, Tokyo Metropolitan Institute of Medical ScienceTokyo, Japan

**Keywords:** corrigendum, Figure 5, cerebral cortex, neuronal migration, molecular pahways, corticogenesis

There are two words missing in the legend of Figure 5 on page 16.

“Acute deletion of RP58 results in MP-BP transition.” should be “Acute deletion of RP58 results in impairment of MP-BP transition.”

## Conflict of interest statement

The authors declare that the research was conducted in the absence of any commercial or financial relationships that could be construed as a potential conflict of interest.

